# Vitellogenin Receptor Mutation Leads to the Oogenesis Mutant Phenotype “*scanty vitellin*” of the Silkworm, Bombyx mori*

**DOI:** 10.1074/jbc.M113.462556

**Published:** 2013-03-20

**Authors:** Ying Lin, Yan Meng, Yan-Xia Wang, Juan Luo, Susumu Katsuma, Cong-Wen Yang, Yutaka Banno, Takahiro Kusakabe, Toru Shimada, Qing-You Xia

**Affiliations:** From the ‡ State Key Laboratory of Silkworm Genome Biology, Southwest University, Chongqing 400716, China,; the § School of Life Sciences, Anhui Agricultural University, 130 West Changjiang Road, Hefei, Anhui 230036, China,; the ¶ Department of Agricultural and Environmental Biology, Graduate School of Agricultural and Life Sciences, University of Tokyo, Yayoi 1-1-1, Bunkyo-ku, Tokyo 113-8657, Japan, and; the ‖ Laboratory of Silkworm Science, Kyushu University Graduate School of Bioresource and Bioenvironmental Sciences, Hakozaki 6-10-1, Fukuoka 812-8581, Japan

**Keywords:** Development, Lipids, Lipid Transport, Receptor Endocytosis, Silkworm, Low-density Lipoprotein Receptor, Mutation, Oogenesis, Vitellogenin Receptor

## Abstract

In insects, the vitellogenin receptor (VgR) mediates the uptake of vitellogenin (Vg) from the hemolymph by developing oocytes. The oogenesis mutant *scanty vitellin* (*vit*) of *Bombyx mori* (Bm) lacks vitellin and 30-kDa proteins, but *B. mori* egg-specific protein and BmVg are normal. The *vit* eggs are white and smaller compared with the pale yellow eggs of the wild type and are embryonic lethal. This study found that a mutation in the *B. mori VgR* gene (*BmVgR*) is responsible for the *vit* phenotype. We cloned the cDNA sequences encoding WT and *vit* BmVgR. The functional domains of BmVgR are similar to those of other low-density lipoprotein receptors. When compared with the wild type, a 235-bp genomic sequence in *vit BmVgR* is substituted for a 7-bp sequence. This mutation has resulted in a 50-amino acid deletion in the third Class B region of the first epidermal growth factor (EGF1) domain. *BmVgR* is expressed specifically in oocytes, and the transcriptional level is changed dramatically and consistently with maturation of oocytes during the previtellogenic periods. Linkage analysis confirmed that *BmVgR* is mutated in the *vit* mutant. The coimmunoprecipitation assay confirmed that mutated BmVgR is able to bind BmVg but that BmVg cannot be dissociated under acidic conditions. The WT phenotype determined by RNA interference was similar to that of the *vit* phenotype for nutritional deficiency, such as BmVg and 30-kDa proteins. These results showed that BmVgR has an important role in transporting proteins for egg formation and embryonic development in *B. mori*.

## Teaser

**Results:** VgR with the mutational EGF1 domain can bind ligand proteins but cannot be dissociated under acidic conditions. The mutant is lethal in embryos.

**Conclusion:** *Bombyx mori* VgR (BmVgR) has an important role in egg formation and embryonic development.

**Significance:** BmVgR is a potential target for pest control.

## Introduction

In insect eggs, vitellin (Vn)^4^ is the most abundant yolk protein (1). The Vn precursor vitellogenin (Vg) is synthesized mainly in the fat body and is transported into growing oocytes by Vg/yolk protein (YP) receptor-mediated endocytosis (VgR/YPR) (2, 3). The processes of synthesis, secretion, and uptake of Vg are important for reproductive development in insects (4). To date, the molecular characteristics and partial functions of VgR/YPR have been identified in 21 insect species (5), and there are reports of the complete cDNA sequence of 11 species (6–15). Moreover, the genomic characteristics of VgR have been obtained from 10 other insect species (16, 17). Insect VgRs belong to the LDL receptor (LDLR) superfamily. All members of this subfamily have similar functional domains, including the ligand-binding domain (LBD), the EGF precursor homology, the *O-*linked sugar domain, the transmembrane domain, and the cytoplasmic domain. Insect VgR transcripts encode a large ovary-specific protein (180∼215 kDa) approximately twice the size of vertebrate VgRs (95∼115 kDa) (6, 9, 11, 18). At neutral pH, LDLRs bind ligands extracellularly, the complex is internalized and then released into the endosomes (pH < 6), leading to lysosomal degradation, and the receptor is recycled to the cell surface (5, 19–24). In addition, LDLRs are involved in lipid metabolism (5). VgR is involved in reproduction, which is a crucial component in the Vg transport mechanism and a potential target for pest control (25, 26).

The domestic silkworm, *Bombyx mori* (Bm) has been used as a Lepidoptera model organism. It synthesizes a large amount of Vg (BmVg), especially in the fat body of female pupae, which is transported into eggs during the pupal stage. However, it is not known whether BmVg is essential for development of the ovaries and embryo in *B. mori*. Yamashita and Irie (27) transplanted silkworm early-stage ovaries into the male silkworm larvae and found that the ovaries developed into mature eggs with chorion and then developed normally into larvae by artificial parthenogenesis. Yamashita and Irie (27) concluded that BmVg is not essential for silkworm egg formation and embryonic development. In contrast, the mutant silkworm strain *vit* (*scanty vitellin*) has a recessive mutation generated by treating eggs with *N*-methyl-*N*-nitrosourea. The resulting eggs are white and smaller compared with normal (WT) pale-yellow eggs, and the homozygote is embryonic lethal. It is suggested that the *vit* mutant could have defective receptors that result in the failure of eggs to take up BmVg and other 30-kDa proteins synthesized outside of the ovary (28–30). Therefore, BmVgR might have a key role in transporting BmVg proteins into developing silkworm eggs. In 2005, the *BmVgR* gene transcript was reported to be a 2564-bp fragment with a poly-A tail (31).

In this study, we compared the morphological features of *vit* eggs and the protein distribution patterns with the *B. mori* WT p50 strain, and BmVg was confirmed to be normal in the *vit* mutant. We cloned the *BmVgR* gene from the wild type and the *vit* mutant to investigate differences in their structure and expression patterns during development of the ovary. Linkage analysis in the *vit* mutant was used to confirm the *BmVgR* mutation. We confirmed by coimmunoprecipitation (co-IP) assay and RNA interference (RNAi) that *BmVgR* mutation resulted in the *vit* mutant. Therefore, BmVgR has a crucial role for egg formation and embryonic development in the silkworm.

## EXPERIMENTAL PROCEDURES

### 

#### 

##### Insects and Tissue Collection

The silkworm *vit* mutant strain (*vit oh*/+ + ♀ × *vit oh/vit oh* 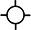) used in this study was obtained from Kyushu University (SilkwormBase). The WT p50 strain was isolated at Kyushu University and is maintained at Southwest University, University of Tokyo, and Anhui Agricultural University. All larvae were reared on fresh leaves of the mulberry tree (*Morus* sp.), and pupae were maintained at room temperature until eclosion.

##### Protein Preparation and Detection

The fat body and ovaries were collected from females at different time points from the start of cocoon spinning to eclosion. Total protein samples were prepared as described (32). Hemolymph specimens were collected simultaneously and treated as described (33). The proteins were subjected to SDS-PAGE (10% (w/v) polyacrylamide gel) according to the method of Laemmli (1970) (34). One gel was stained with Quick Coomassie brilliant blue (CBB) (Wako, Osaka, Japan) and photographed using the LAS 1000 imaging system (Fuji Film). Another gel was analyzed by Western blot analysis. The proteins in the gel were transferred electrophoretically onto a PVDF membrane (Roche) that was blocked by 5% (v/v) nonfat milk at 4 °C overnight. The membrane was incubated with α-tubulin antibody (1:10,000) (Beyotime, China) and anti-BmVg polyclonal rabbit antibody (1:10,000) at room temperature for 2 h. (Recombinant antigen BmVg (amino acid positions 700–1053) is expressed with the pET28a-BmVg plasmid in *Escherichia coli* strain BL21 (DE3) (TransGen Biotech, France). Antigen protein was purified using metal-affinity resin (GE Healthcare). This antigen protein was injected into rabbits for antibody production. Anti-BmVg polyclonal antibody was purified using antigen-affinity resin.) The membrane was washed three times for 10 min each time. The membrane was incubated with HRP-labeled goat anti-rabbit IgG (H+L) (1:50,000) (Beyotime) at room temperature for 60 min and washed three times for 10 min each time. The bound HRP-conjugated antibodies were displayed by an enhanced chemiluminescence system (Thermo) and photographed using the ChemiScope 3400 Mini (Clinx Science Instruments, China).

##### Total RNA Extraction and cDNA Synthesis

Total RNA was extracted using a TRIzol reagent kit (Invitrogen), and molone-murine leukemia virus (M-MLV) reverse transcriptase (Invitrogen was used to generate the first-strand cDNA. Each kit was used according to the instructions of the manufacturer.

##### Cloning and Sequencing

Degenerate primers were designed on the basis of the coding sequence (CDS) sequence of *BmVgR* (*BGBMGA*014160-4) and AY676608 downloaded from the silkworm genome database, SilkDB v2.0 (35). Five pairs of primers were designed, and the PCR fragments had overlapping regions to enable accurate splicing. To include the 5′ UTR of *BmVgR*, the primers were selected between the transcriptional start site and the eighth exon site. The resulting cDNA fragment was PCR-amplified using En-HiFi TaqDNA polymerase (TransGen Biotech). The PCR conditions were 94 °C for 5 min, then 30 cycles at 94 °C for 30 s, 55–60 °C for 30 s, 72 °C for 1 min/kb, and, finally, extension at 72 °C for 10 min. The PCR products were ligated into the pMD19-T vector (TaKaRa Biotech, Japan) and transformed into *E. coli* DH5α^TM^-competent cells according to the instructions of the manufacturer (TransGen Biotech). Positive clones were confirmed by electrophoresis and sequenced by Invitrogen.

##### Sequence Analysis of the BmVgR Gene

The exon/intron structures of *BmVgR* were determined using sim4 software (36). The isoelectric point and the molecular mass were predicted by the Compute pI/Mw tool (37). The signal peptide position was predicted using Signal P software (38). VgR/YPR sequences of other species were aligned with BmVgR using Clustal X software (39). The amino acid sequences used for comparison were downloaded from the National Center for Biotechnology Information.

##### Semi-quantitative RT-PCR

The antennae, head, thorax, abdomen, wings, testes, ovaries, fat body and pheromone glands were dissected from *B. mori* strain p50 adults. Whole bodies were dissected at 12 different time points from embryo to third instar larva, and plant material was removed from the intestine. Ovaries were dissected at an additional 23 different time points, from fourth instar larva to adult. All tissues and unfertilized eggs collected at three different time points were frozen immediately in liquid nitrogen and then stored at −80 °C. Total RNA extraction and cDNA synthesis followed the process described above. The PCR primers were as follows: BmVgR-EF, 5′CGAAGAACTGCGAGACCTACAT3′ and BmVgR-ER 5′TGGGCACTGCTATTGACAGG3′. The PCR conditions were 94 °C for 5 min, then 25 cycles at 94 °C for 30 s, 60 °C for 30 s, and 72 °C for 1 min, and, finally, extension at 72 °C for 10 min. PCR products were confirmed by electrophoresis in 1% (w/v) agarose gel and photographed by the Molecular Imager Gel Doc XR system (Bio-Rad). The *Bmactin3* gene was used as the control.

##### In Situ Hybridization

To detect the expression patterns of *BmVgR* using *in situ* hybridization, a 570-bp fragment was amplified to generate RNA probes using a bacteriophage T7 sequence as the adaptor. The PCR primers used in the experiments were as follows: T7-BmVgR-SF1, 5′GTAATACGACTCACTATAGGGAGACGAAGAACTGCGAGACCTACAT3′ and BmVgR-SR1 5′TGGGCACTGCTATTGACAGG3′; BmVgR-SF2 5′CGAAGAACTGCGAGACCTACAT3′ and T7-BmVgR-SR2 5′GTAATACGACTCACTATAGGGAGATGGGCACTGCTATTGACAGG3′. Probes were synthesized according to the instructions accompanying the digoxigenin (DIG) RNA labeling kit (SP6/T7) (Roche). The efficiency of the probe was determined by spot blots on nylon membranes with anti-DIG-alkaline phosphatase Fab fragments (Roche). Fresh p50 strain ovarian tissues were fixed in 4% (w/v) paraformaldehyde at 4 °C for 4 h, dehydrated, embedded in paraffin, and cut into 5- to 7-μm-thick samples by a slicing machine (Leica Rm2235, Germany). The slices were allowed to adhere to glass slides and then incubated at 42 °C for 12 h. Samples were washed with xylene twice, dehydrated slowly, washed with 0.1% (v/v) diethylpyrocarbonate-treated water, and then prehybridized in hybridization solution at 42 °C for 30 min. Each section was hybridized at 45 °C for 16 h with a DIG-labeled RNA probe, detected with anti-DIG-alkaline phosphatase Fab fragments, and visualized using nitro blue tetrazolium/5-bromo-4-chloro-3-indolyl phosphate solution (Roche). Finally, stained slices were rinsed several times with deionized water and observed under a DX50 microscope (Olympus, Melville, NY).

##### Northern Blotting

Total RNA was extracted from ovaries of *B. mori* d50 (*vit/vit*) and p50 (WT) strain pupae on days 4 and 6, respectively, as described (33). A total of 5 μg of RNA was resolved by electrophoresis in 1.2% (w/v) agarose gels containing 2.2 m formamide in MOPS buffer and transferred electrophoretically onto a Hybond XL nylon membrane (Amersham Biosciences, UK). The membrane was hybridized with two probes synthesized using the plasmid DNA containing WT *BmVgR* cDNA as a template by a DIG DNA labeling and detection kit (Roche). One probe was designed for the detection of both types of *BmVgR* RNA and the other only for WT RNA. DIG Easy Hyb granules, blocking reagent, anti-DIG-alkaline phosphatase conjugate, and disodium 2-chloro-5-(4-methoxyspiro{1,2-dioxetane-3,2′-(5′-chloro)tricyclo[3.3.1.1^3,7^]decan}-4-yl)-1-phenyl phosphate (CDP) Star (Roche) were used according to the instructions of the manufacturer. The blot was visualized using the LAS 1000 imaging system (Fuji Film), and the *Bmrp49* gene was used as the control. The primers used for probe synthesis were as follows: BmVgR-P1F, 5′CACAGCCTCCTGTCCCAC TCGATGC3′, BmVgR-P1R, 5′CAGTTGGCGC AGAAGGGTCTCTC3′; BmVgRP2, 5′CCCAGCGTTGATGAAGAAGATTCC3′; and BmVgR -P2R, 5′GGATGCATCTGCCGTTCTT GTT3′.

##### Quantitative RT-PCR

Quantitative reverse transcription PCR (qRT-PCR) was done using SYBR Premix ExTaqTM (TaKaRa Biotech, Japan) and the ABI Prism 7000 sequence detection system (Applied Biosystems) to determine the *BmVgR* mRNA level in d50 and p50 strains quantitatively and to evaluate the effect of *BmVgR* RNAi in the p50 strain. The *BmTIF4A* gene was used as an endogenous control. The qRT-PCR primers used in the experiment were as follows: BmVgR-qF 5′GAGTGCCTGGGCGAGGATGT3′; BmVgR-qR, 5′CTGAGCGTCTGGCTTGTGA3′.

##### Linkage Analysis

The *vit* mutant strain, which is maintained by mating *vit oh*/+ + females with *vit oh/vit oh* males, was used in the genetic linkage analysis. Heterozygous moths in the F_1_ progeny were sibling-mated to produce recombinant individuals between +*^vit^* and *vit*. After oviposition, genomic DNA of 266 female moths was isolated using DNAzol® reagent (Invitrogen) according to the instructions of the manufacturer, and we monitored whether the eggs hatched. Genotypes of female moths were determined by a PCR marker containing a 228-nt genomic deletion in the mutant type of *BmVgR*. The PCR primers were as follows: BmVgRLF, 5′CACTTGACATGAGATGAGCA GTGA3′; BmVgRLR, 5′TCATAGGTCATAGC TTGCACGCGT3′.

##### Co-IP Assay

The co-IP procedure followed the instruction for the kit (Invitrogen), and cell expression vectors were constructed. The cDNA of *BmVgR* p50 and d50 strains were used as templates for PCR amplification of BmVgR-LBD1+EGF1 (p50, D11; d50, V11) with a *Myc*-tag and the signal peptide. The 5′ primer sequence contained a BglII restriction site. The 3′ primer sequence contained a *Myc*-tag, a stop codon, and a NotI restriction site. The PCR products of 2790 bp and 2640 bp were subsequently cloned into the *pEASY*-T1 simple cloning vector (TransGen Biotech) and then digested with BglII and NotI (TaKaRa Biotech). The target fragments were obtained by gel purification and then cloned into the BamHI- and NotI-digested 1180 [Hrs1000-BmAct4-LUC-Ser1PA] expression vector (maintained in our laboratory). D11 and V11 were expressed in sf9 cells (the *Spodoptera frugiperda* ovary cell), which do not express endogenous *BmVg* and *BmVgR* genes (data not shown). Highly purified plasmid DNA was prepared using the Qiagen plasmid midi kit (Qiagen, Germany). After transfection for 72 h, the culture medium was collected by centrifugation (500 × *g* at 4 °C for 5 min). The culture medium was tested by Western blotting with anti-Myc mouse antibody. A 200-μl sample of purified BmVg (0.02 μg/μl) (measured by the Bradford method) (40) from silkworm pupal hemolymph was added into the culture medium and incubated at room temperature for 90 min to obtain the D11/V11-BmVg complex. Then, 8.4 μg of anti-Myc mouse antibody (Invitrogen) diluted in 200 μl of culture medium was added to 50 μl (1.5 mg) of 5% (w/v) BSA-blocked Dynabeads (Beyotime) and incubated with rotation for 10 min at room temperature. The supernatant was collected by a magnet and retained. The beads-Ab complex was washed twice in 200 μl of culture medium. The beads-Ab cross-linked complex was then added to the D11/V11-BmVg complex in a tube and incubated with rotation for 90 min at 4 °C. The supernatant was transferred into a clean tube, and the beads-Ab-D11/V11-BmVg complex was washed in 200 μl of culture medium and then washed in 200 μl of PBS (137 mmol/liter NaCl, 2.7 mmol/liter KCl, 10 mmol/liter Na_2_HPO_4_, and 1.8 mmol/liter KH_2_PO_4_ (pH 7.4)). The pH of the *vit* mutant egg was 5.56, and that of the normal p50 strain egg was 5.85. Therefore, the complex was divided into two equal portions. One was washed three times with 200 μl of PBS (pH 7.4), and the other was washed three times using the same volume of PBS (pH 5.5). The complex was suspended in 100 μl of PBS (pH 7.4) and transferred into a clean tube. Then, 20 μl of elution buffer (50 mm glycine (pH 2.8)), 10 μl of premixed NuPAGE lithium dodecyl sulfate (LDS) sample buffer, and NuPAGE sample reducing agent (Invitrogen) were added, and the mixture was heated for 10 min at 100 °C. The tube was placed onto a magnet, and the eluate from the beads was subjected to SDS-PAGE (8% (w/v) polyacrylamide gel) and then analyzed with anti-BmVg polyclonal rabbit antibody (1:5000) and HRP-labeled goat anti-rabbit IgG (H+L) (1:40,000) (Beyotime) by Western blot analysis as described above.

##### Gene Silencing

A clone of *a BmVgR* segment was used as a template to generate the 695-bp fragment dsBmVgR (amino acid positions 552–783), which was located in the EGF1 domain. A bacteriophage T7 promoter sequence was used as an adaptor, and dsRed was used as the control in the RNAi experiments. All double-stranded RNAs were synthesized using a RiboMAX large-scale system T7 kit (Promega, Madison, WI) following the instructions of the manufacturer, and the resulting double-stranded RNA was stored at −80 °C. Then, p50 strain female pupae on the first pupal day were injected with 40 μg of dsBmVgR by capillary needle into the intersegmental membrane between the eighth and ninth abdominal segment. To achieve a better effect, the pupae were injected again on days 4 and 7 with the same dose. Water and dsRed were injected at the same time, and the same dose was used for the controls. The treated pupae were maintained at room temperature. After eclosion, some injected double-stranded RNA female moths were mated with untreated males and laid eggs. The other female moths were dissected to observe ovarian development.

##### ELISA

Total ovarian protein was extracted when double-stranded RNA-treated pupae reached eclosion. The amount of protein was quantified by the Bradford method (40). ELISA detection with the insect Vn ELISA kit (R&D Systems) was done with an immunowash apparatus (model 1575, Bio-Rad) according to the instructions of the manufacturer.

## RESULTS

### 

#### 

##### Morphological Characteristics of vit Mutant Eggs and Distribution of Proteins

**FIGURE 1.**
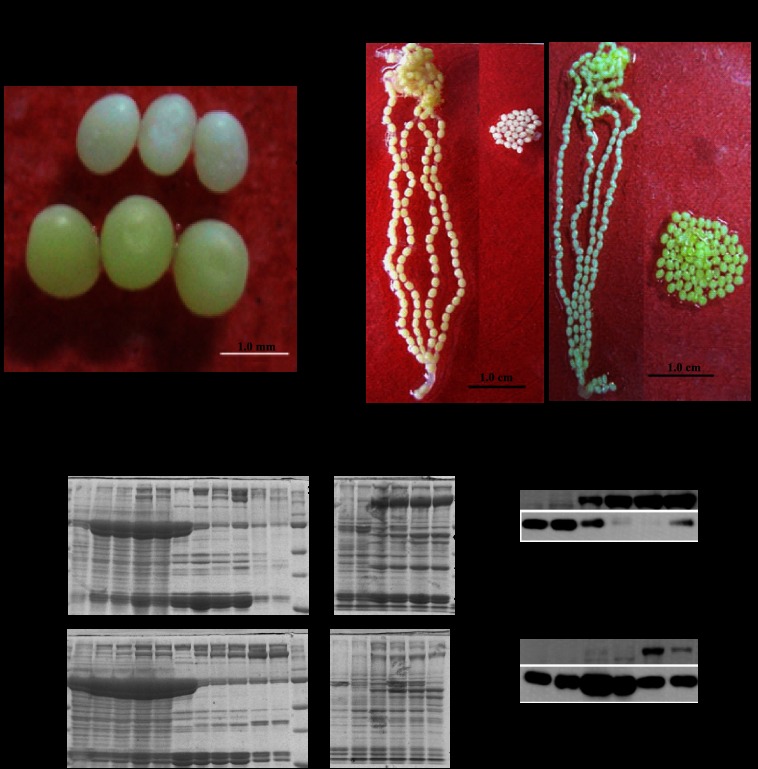
**The morphological characteristics of eggs and the protein distribution patterns of the *vit* mutant.** *A*, morphological characteristics of d50 *vit* mutant eggs were compared with those of WT strain p50 eggs. The eggs were unfertilized and were photographed on the day of eclosion. *B*, protein analysis of the *vit* mutant. *B1*, analysis of total proteins from the fat body, hemolymph, and ovaries of female moths by SDS-PAGE. *B2*, analysis of the Vn protein of the ovary by Western blot analysis. *30K*, 30-kDa plasma proteins; *SP*, storage proteins; *H*, heavy subunit; *L*, light subunit; *S*, spinning; *L-P*, larva-pupa; *P*, pupa (numbers are days); *M_0_*, moth; *M*, standard protein markers. *C*, ovary transplant. *C1*, transplant of the early pupa ovary of the *vit* mutant (donor) into the normal early female silkworm pupa (acceptor); *C2,* Transplant the early pupa ovary of the normal silkworm (donor) into the early female *vit* mutant pupa (acceptor). *C1* and *C2*, eggs of the acceptor (*left panel*) and eggs of the donor (*right panel*). The white eggs laid by *vit* homozygous moths were smaller compared with the pale yellow WT eggs. Eggs of the *vit* mutant have fewer Vn and 30-kDa proteins than the wild type but a normal amount of egg-specific protein, and BmVg function is normal in the *vit* mutant.

Earlier studies showed that newly oviposited eggs of the *vit* mutant are white and lack Vn and 30-kDa proteins, although the precursors of these proteins are abundant in the pupal hemolymph (28, 29, 41). We compared the morphological characteristics of eggs and the protein distribution patterns of d50 (*vit/vit*) to those of the WT p50 strain. Consistent with the results of earlier studies, the eggs laid by *vit* homozygous moths were white and smaller compared with WT eggs (Fig. 1*A*). SDS-PAGE showed that there was no significant difference between the d50 and p50 strains in either the synthesis of Vg and 30-kDa proteins in the fat body of female pupae or the secretion of the two proteins into hemolymph until the second day (*P2*) (Fig. 1, *B1*). However, after the P2 stage, the majority of Vg and 30-kDa proteins in the d50 strain remained in the hemolymph rather than being taken up by the ovaries. In contrast, most of the BmVg and 30-kDa proteins in strain p50 were absorbed by the ovaries and stored as BmVn (Fig. 1, *B1*). This result was confirmed by Western blot analysis (Fig. 1, *B2*). As a comparison, *B. mori* egg-specific protein (*BmESP*), which is produced only by the ovary, was normal in the *vit* mutant (Fig. 1, *B1*). We had transplanted an early pupal ovary of the *vit* mutant into a normal early female silkworm pupa and an early pupal ovary of a normal silkworm into an early female *vit* mutant pupa. The results showed that the ovary of the *vit* mutant develop from *vit* eggs in the WT silkworm pupa (Fig. 1, *C1*). The normal ovary can develop and form normal eggs in the pupa of the *vit* mutant (Fig. 1, *C2*), suggesting that BmVg is normal in the *vit* mutant and that corresponding receptors should be defective.

##### Cloning and Structural Analysis of BmVgR

**FIGURE 2.**
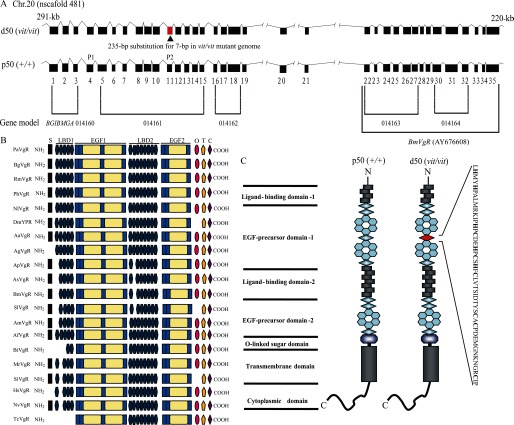
**Sequence and structural analysis of *BmVgR* of WT and the *vit* mutant strains.** *A*, cloning of complete *BmVgR* cDNA and the gene structure alignment between d50 (*vit/vit*) and p50 (WT) strains. Total RNA was extracted from ovaries of d50- and p50-strain moths on the day of eclosion. Degenerate primers were designed on the basis of the coding sequence (CDS) sequence of *BmVgR* (*BGBMGA*014160-4), AY676608, and genomic sequence (nscafold481) downloaded from the silkworm genome database, SilkDB v2.0. *P*, probe (numbers are order number). *B*, schematic alignment of modular domains from BmVgR and other insects VgR/YPR. *P. americana* VgR (PaVgR, BAC02725), *B. germanica* VgR (BgVgR, CAJ19121), *Rhyparobia maderae* VgR (RmVgR, BAE93218), *Pediculus humanus* corporis VgR (PhVgR, EEB10383), *Nilaparvata lugens* VgR (NlVgR, ADE34166), *D. melanogaster* YPR (DmYPR, AAB60217), *Aedes aegypti* VgR (AaVgR, AAK15810), *Anopheles gambiae* VgR (AgVgR, EAA06264), *A. pernyi* VgR (ApVgR, AEJ88360), *A. selene* VgR (AsVgR, AFV32171), *B. mori* VgR (BmVgR, ADK94452), *S. litura* VgR (SlVgR, ADK94033), *A. mellifera* VgR (AmVgR, XP_001121707), *Apis florae* VgR (AfVgR, XP_003690500), *Bombus impatiens* VgR (BiVgR, XP_003489577), *Megachile rotundata* VgR (MrVgR, XP_003704660), *S. invicta* VgR (SiVgR, AAP92450), *Harpegnathos saltator* VgR (HsVgR, EFN84770), *Nasonia vitripennis* VgR (NvVgR, XP_001602954), and *Tribolium castaneum* VgR (TcVgR, XP_968903). *S*, signal peptide; *LBD*, class A (complement-type) Cys-rich repeats; *EGF*, class B Cys-rich (EGF-type) repeats; *O*, *O-*linked sugar domain; *T*, transmembrane domain; *C*, cytoplasmic tail. The functional domains of BmVgR are similar to the VgR/YPR of other insect species but differ in having four class A repeats in LBD1 and seven class A repeats in LBD2, which might be unique to Lepidoptera. *C*, predicted structural organization of *BmVgR* with reference to Tufail *et al* (5). BmVgR has five modular domains, similar to other LDLR family members. It harbors two of the LBD and EGF domains and has four class A repeats in LBD1 and seven class A repeats in LBD2. A deletion of 50 amino in the *vit* mutant compared with WT (shown in *red*), which belong to the third class B region of the EGF1 domain of BmVgR.

Physical mapping of *BmVgR* indicates that it is located on chromosome 20 (35), which is consistent with genetic mapping of the *vit* gene (42). This suggests that the *BmVgR* gene might be the *vit* gene. Therefore, we cloned and analyzed the *BmVgR* gene, which spans > 70 kb in the genome, with ∼35 exons, and contains a complete open reading frame (Fig. 2*A*). The mature BmVgR protein contains a signal peptide (MKVVLLAIVLCTTSCVG), as predicted by the Signal P software (38), with a cleavage site between amino acid positions 17 (Gly) and 18 (Gln). The molecular mass and pI of BmVgR before removal of the signal peptide were 202.5 kDa and 5.54 kDa, respectively, as predicted by the Compute pI/Mw tool (37). After removal of the signal peptide, the mature protein had a predicted molecular mass of 200.8 kDa with pI 5.52. BmVgR contains two LBDs with four class A repeats in the first LBD (LBD1) and seven repeats in the 2nd LBD (LBD2). Each class A repeat is composed of six Cys repeats and an Ser-Asp-Glu (SDE) amino acid cluster, which might be involved in secondary or tertiary structure formation (43). In addition to the LBD, there are EGF precursor homology domains that contain six Cys repeats (class B) and YWXD motifs. Class B repeats are involved in receptor dimerization and receptor-ligand dissociation (44, 45), and the YWXD motif is required to form the propeller, which is usually found in molecules involved in protein-protein interactions (25). A putative *O-*linked sugar domain at amino acid positions 1661–1688 had four Ser and two Thr residues. A hydrophobic region that might function as a membrane anchor from amino acid positions 1688–1710 was predicted to be the transmembrane domain using the Expasy TMHMM server v. 2.0. The cytoplasmic domain, which was predicted to be from amino acid positions 1711–1816, contains one copy of the NPLQ and LI motifs and possibly functions as an internalization signal to form coated pits in the silkworm. BmVgR has the highest level of homology with AsVgR and ApVgR, with 98% amino acid identity. SlVgR has 56% amino acid identity but only ∼31% amino acid identity with VgRs from the other insect species examined (Fig. 2*B*). In terms of its genomic sequence, a 235-bp fragment of *BmVgR* from the d50 strain is substituted for a 7-bp fragment, which results in a loss of the 11th exon and 50 amino acids from the protein sequence (Fig. 2*A*). Structural analysis showed that this deletion is in the third class B region of the EGF1 domain (Fig. 2*C*). This suggests that BmVgR of the *vit* mutant can bind ligands such as BmVg or 30-kDa proteins but cannot be dissociated under acidic conditions.

##### Expression Patterns and Cell Localization of BmVgR

**FIGURE 3.**
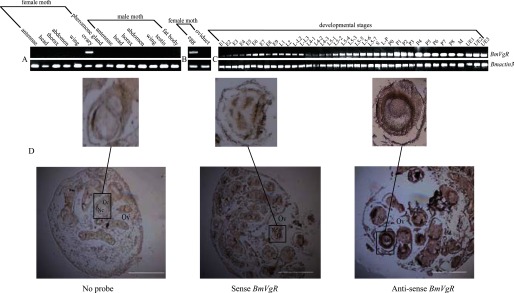
**Expression patterns of *BmVgR* in the WT strain.** *A–C*, expression profiles of WT *BmVgR* in different sex, tissue, and development stages. The total RNA in *A* and *B* samples was isolated from the antennae, head, thorax, abdomen, wings, ovaries, pheromone glands, eggs, and oviducts of p50-strain (WT) female moths and antennae, head, thorax, abdomen, wings, testes, and fat body from p50-strain male moths. The total RNA in *C* was from samples of whole p50-strain individuals from the embryo stage to the third instar larva and then from various tissues from the fourth instar larva to eclosion. *E*, embryo; *S*, spinning; *L-L*, molting; *L-P*, larva-pupa; *P*, pupa (numbers are days); *M*, moth; *UE*, unfertilized egg. Amplification of the *Bmactin3* gene was used as an endogenous control. *BmVgR* was expressed specifically in ovarian tissue and restricted to eggs but was not expressed in the oviduct. It was expressed throughout the life span of the silkworm and was up-regulated during egg maturation. *D*, the *BmVgR* transcript detected in WT oocytes by *in situ* hybridization. *Nc*, nurse cell; *Oc*, oocyte; *Ov*, ovary. There was a detectable expression only in the oocytes. *Scale bar* = 50 μm.

RT-PCR analysis of different tissues in p50 adults showed that *BmVgR* is expressed specifically in ovarian tissue and restricted to the eggs (Fig. 3, *A* and *B*). RT-PCR analysis demonstrated *BmVgR* is expressed throughout the lifespan of the silkworm, as shown in Fig. 3*C*. However, the expression level of *BmVgR* was low from the embryo stage to day 5 of the fifth instar larva and began to increase on day 6 of the fifth instar larva, reaching the highest level on day 4 after pupation. To investigate which part of the ovary expressed *BmVgR*, ovaries from day 1 after pupation were probed with fragments of *BmVgR*. Oocytes were stained blue-purple by the antisense probe, and no signal was detected using water and a sense probe, indicating that *BmVgR* is expressed only in oocytes (Fig. 3*D*).

##### Transcriptional Expression Analysis of BmVgR

**FIGURE 4.**
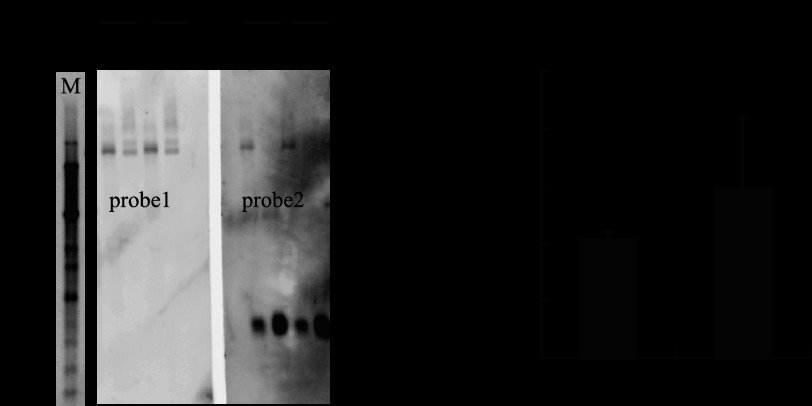
**Expression patterns of *BmVgR* in WT and *vit* mutant strains.** *A*, Northern blot analysis. Total RNA (5 μg) was collected from ovaries of d50-strain (*vit/vit*) and p50-strain (WT) pupae on days 4 and 6, respectively. Two kinds of cDNA probes were synthesized, as shown in Fig. 2. *M*, standard RNA marker; *P*, pupa (numbers are days). The *Bmrp49* gene was used as the control. *B*, qRT-PCR. *BmVgR* mRNA transcription in ovaries on the day of eclosion was determined quantitatively using the *BmTIF4A* gene as an endogenous control. Analysis of variance for three independent experiments was done with MS Excel 2003 software. All statistical analyses were done with SPSS v. 15.0 software (Chicago, IL. *BmVgR* was transcribed in *vit* individuals, but its expression level was not reduced significantly.

Northern blot analysis and qRT-PCR detected the transcriptional level of the *BmVgR* gene in the d50 and p50 strains using two probes, one from the common exon 4 and one from exon 11, which is deleted from *BmVgR* of the *vit* mutant (Fig. 2). For the d50 and p50 strains, a single transcript of 5.6 kb corresponding to the predicted molecular size of *BmVgR* mRNA was detected in the ovary when probe 1 was used. In contrast, only the p50 strain *BmVgR* transcript was detected when probe 2 was used (Fig. 4*A*). These results confirmed the gene identification and characterization described above (Fig. 2). qRT-PCR also showed the relative expression level of *BmVgR* mRNA in the d50 strain was lower than that in the p50 strain, but the difference was not statistically significant, as measured by three independent experiments (Fig. 4*B*).

##### Linkage Analysis between vit and the BmVgR Gene

**FIGURE 5.**
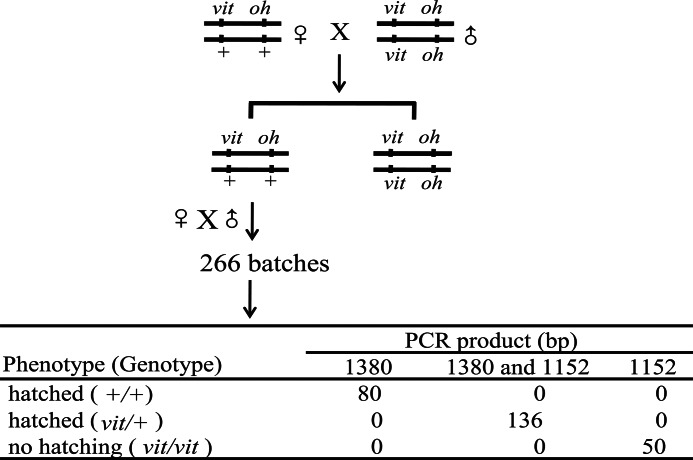
**Linkage analysis between *vit and BmVgR*.** With the *oh* phenotype in the d50 strain (*vit/vit*), *vit*/+ female moths were selected and crossed to *vit/vit* male moths. Their non-oily offspring were sibling-mated to produce recombinant individuals. The genomic DNA of 266 F_1_ female moths was isolated and used in the linkage analysis. The hatching phenotype of oviposited eggs was observed. The 228-bp genomic deletion in the *vit* mutant *BmVgR* gene was used as a PCR marker. The results indicate that the mutation of *BmVgR* is responsible for the *vit* mutant phenotype.

A linkage analysis was done with F_1_ progeny of the d50 strain to elucidate the relationship between the *vit* phenotype and the *BmVgR* genotype. With the help of *hoarfrost translucent* (*oh*), a recessive *oily* gene that causes moderately translucent mottling of the larval skin with several indistinct fine opaque dots (28, 46), *vit*/+ heterozygous larvae could be differentiated from *vit/vit* homozygous larvae. After eclosion, *vit*/+ female and male moths were crossed (Fig. 5). The hatching ability of 266 batches of eggs was evaluated. The genomic DNA of the mothers was extracted for genetic linkage analysis by a PCR marker of *BmVgR* (+/+, 1380 bp, *vit/vit*, 1152 bp). As expected, mothers of all the lethal eggs were homozygous for a single 1152-bp band, whereas other moths, whose eggs hatched normally, were homozygous for a single 1380-bp band or heterozygous for two bands (1380 bp and 1152 bp) (Fig. 5). These results suggest that there is no recombination between the *vit* locus and *BmVgR* genes. On the basis of these results, we concluded that the candidate gene *BmVgR* corresponds to the *vit* gene.

##### Ligand-Receptor Interactions

**FIGURE 6.**
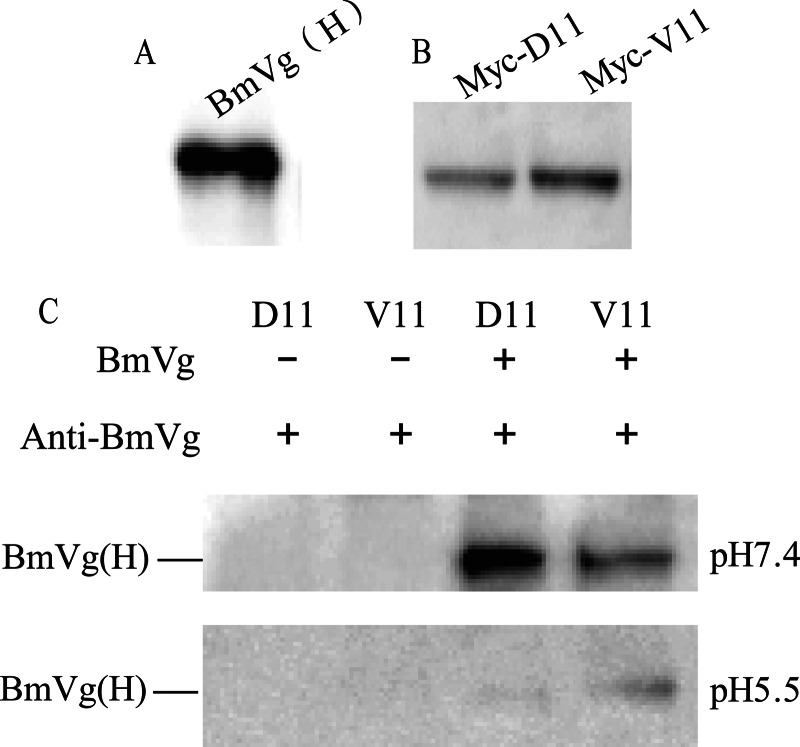
**Binding and dissociation between BmVg and BmVgR by co-IP assay.** *A*, BmVg (H). The large subunit of purified BmVg (180 kDa) from the silkworm pupa hemolymph was recognized by anti-BmVg polyclonal rabbit antibody. *B*, expression of normal and mutational BmVgR-LBD1+EGF1 (p50, D11; d50, V11) with a *Myc* tag and the signal peptide in sf9 cells were detected by anti-Myc antibody. *C*, in the co-IP assay, Myc-D11 and Myc-V11 were incubated with BmVg at room temperature and then coupled to Dynabeads® protein G with the anti-Myc antibody. The beads-Ab-D11/V11-BmVg complex was washed in culture medium and then washed in PBS (pH 7.4). The complex was divided into two equal portions. One was washed three times using PBS (pH 7.4), and the other was washed three times using PBS (pH 5.5). The unbound proteins were washed away, and the bound proteins were heated for 10 min in boiling water. Then, BmVg was detected with anti-BmVg polyclonal rabbit antibody by Western blotting. Myc-D11 and Myc-V11 (without BmVg) were used as the negative control. These results show that the BmVgR of the *vit* mutant can bind BmVg protein but cannot be dissociated in the acidic environment.

Purified BmVg from silkworm pupa hemolymph was detected by Western blotting with anti-BmVg polyclonal rabbit antibody (Fig. 6*A*). Expression of normal and mutational BmVgR-LBD1+EGF1 (p50, D11; d50, V11) with a *Myc* tag and the signal peptide in sf9 cells could be detected by Western blotting with anti-Myc mouse antibody (Fig. 6*B*). Therefore, we detected Myc-D11 and Myc-V11 interactions by co-IP assay, which showed that Myc-D11 and Myc-V11 can bind BmVg at neutral pH (7.4) (Fig. 6*C*). BmVg can be dissociated from the Myc-D11 but not from the Myc-V11 at acidic pH (5.5) (Fig. 6*C*). These results showed that the BmVgR of the *vit* mutant, which is mutated in the third class B region of the EGF1 domain, can bind ligands such as BmVg and 30-kDa proteins. However, ligands cannot be dissociated from mutational BmVgR in the acidic environment of the silkworm egg (*vit* mutant, pH 5.56; p50 strain, pH 5.85). The *vit* mutant is lethal for nutritional deficiency.

##### RNAi of BmVgR

**FIGURE 7.**
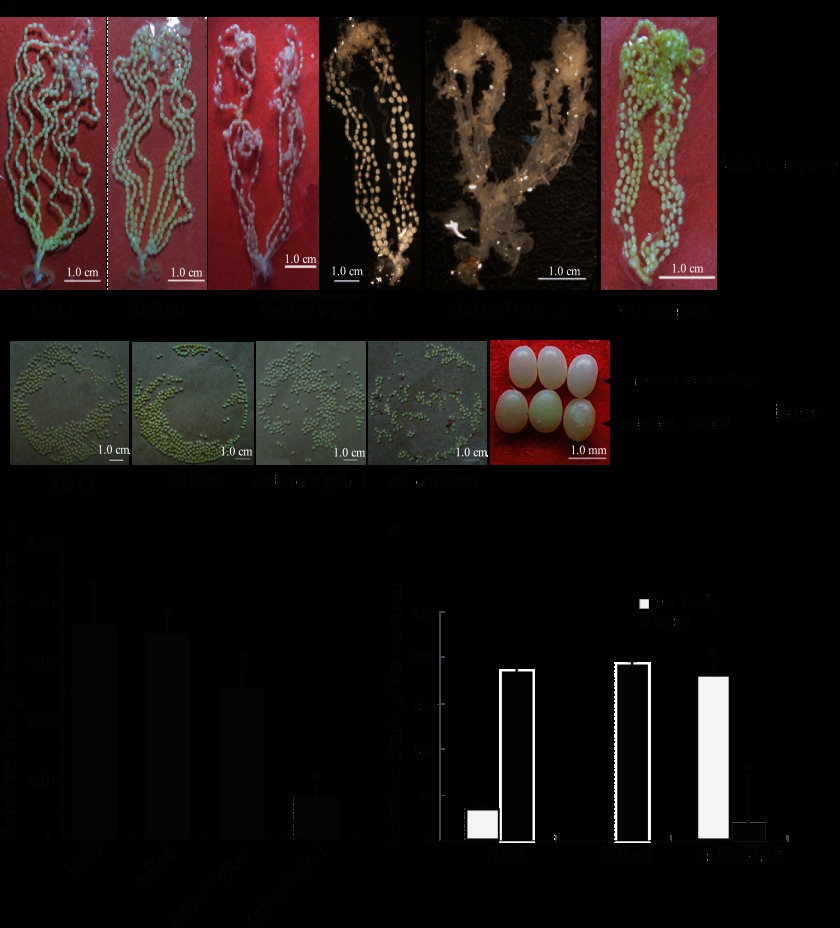
**RNA interference of *BmVgR* in female silkworm pupae.** An equal volume of dsBmVgR, dsRed, or water was injected separately into ten WT p50-strain female pupae. *A*, anatomy of the injected female pupae and the phenotype of eggs injected with water, dsRed, or dsBmVgR. The phenotype of three females injected with dsBmVgR as pupae was similar to that of the *vit* mutant, laying smaller, white eggs that did not hatch. Six females injected with dsBmVgR as pupae did not have eggs in the oviducts. *B*, the relative *BmVgR* mRNA level in ovaries of dsBmVgR treated on the day of eclosion was determined by qRT-PCR analysis using the *BmTIF4A* gene as the endogenous control. *C*, ELISA was used to measure the content of BmVn/Vg in the eggs and the fat body of dsBmVgR-treated moths. Analysis of variance for three independent experiments was done with MS Excel 2003 software. Statistical analysis was done with SPSS v.15.0 software. Differences between dsRed and dsBmVgR were considered significant (*, *p* < 0.05). The relative BmVgR mRNA level of dsBmVgR-treated eggs was significantly lower than that of moths treated with water or dsRed. The content of BmVn in dsBmVgR-treated eggs was lower than that of eggs treated with water or dsRed, whereas BmVg in dsBmVgR-treated fat bodies was more abundant than that in fat bodies treated with water or dsRed.

On the basis of the *BmVgR* expression patterns, we chose ten p50-strain female pupae on the first day of pupation for dsBmVgR injection. Three of them reached eclosion and mated normally but laid white eggs that were smaller than the pale yellow eggs laid by adults from the control pupae injected with either water or dsRed (Fig. 7*A*, *dsBmVgR-1*). The phenotype was similar to that of the *vit* mutant, especially in terms of the size and color of the eggs (Fig. 7*A*), and small eggs did not hatch. In addition, six of them reached eclosion and mated normally but failed to lay eggs, and there was no egg in the oviduct (Fig. 7*A*, *dsBmVgR-2*). The remaining one appeared normal. In contrast, all control female pupae injected with water or dsRed developed normally and laid normal eggs (Fig. 7*A*).

qRT-PCR revealed that the mRNA levels of *BmVgR* in pupae treated with dsBmVgR were reduced and that the pupae that produced no egg (dsBmVgR-2) expressed lower levels of *BmVgR* mRNA than those that produced small eggs (dsBmVgR-1) (Fig. 6*B*). ELISA results showed that the amount of BmVn in the small eggs laid by pupae treated with dsBmVgR was lower than that of controls, whereas the content of BmVg in the fat body of the adults was higher than that of the controls (Fig. 7*C*), indicating that, after RNAi treatment, BmVg could not be transported normally into oocytes by receptor-mediated endocytosis because of the deficiency in BmVgR. This result was similar to that of the phenotype of the *vit* mutant for nutritional deficiency (Fig. 1*A*).

## DISCUSSION

A histological study of the *vit* mutant showed that its ovarian follicle cells and the gaps between them are morphologically normal (29). Our study found that precursors of Vn and 30-kDa proteins from the hemolymph are not normally absorbed into *vit* mutant oocytes and that the eggs of the *vit* mutant have fewer Vn and 30-kDa proteins compared with WT but a normal amount of egg-specific protein. In addition, the BmVg function of the *vit* mutant is normal, and physical mapping of *BmVgR* is consistent with genetic mapping of the *vit* gene, which is located on chromosome 20 (35). Thus, it was speculated the *vit* mutant phenotype is caused by a defective receptor. We suggested, on the basis of the predicted functions of different regions and protein distribution patterns, that the defective BmVgR protein in the *vit* mutant might be caused by the deletion of crucial domains. The mutation could be the reason why BmVgR cannot be dissociated from BmVg and 30-kDa proteins in the *vit* mutant egg. Therefore, BmVgR could not recycle or promote rapid degradation of the Vg-VgR complex (5, 44, 47), causing a lack of accumulation of Vn and 30-kDa proteins in the eggs of the *vit* mutant.

In this study, we showed that the functional domains of BmVgR are similar to those of VgRs/YPR in other insect species (Fig. 2*B*). In particular, there is a striking homology between BmVgR and the VgR of *Actias selene*, *Antheraea pernyi*, and *Spodoptera litura*, which belong to the Lepidoptera. This could be the result of the high degree of similarity of their ligands. Evolutionary conservation between BmVgR and other insect VgRs/YPR suggests that BmVgR is a member of the LDLR family bearing five highly conserved arrangements of modular elements (Fig. 2*B*). The four class A repeats in the LBD1 of BmVgR are similar to those in the VgR of *A. selene, A. pernyi*, *S. litura*, and *Apis mellifera* (8, 12, 14, 15). The second class A region in LBD2 contains only seven repeats in *B. mori*, *A. pernyi*, *A. selene*, and *S. litura,* and this might be unique to the Lepidoptera. The class B region is essential for VgR functions. In ligand dissociation and receptor recycling assays, Davis *et al.* (44) showed that deletion of the three Cys repeat regions in the EGF domain can affect the function of LDLR. The mutated receptor in this region cannot be dissociated from ligands under acidic conditions and is degraded shortly after ligand binding. Thus, it cannot be recycled effectively. His-562 and His-586 in the EGF domain of human LDLR are important for ligand-receptor dissociation (47). The BmVgR of the *vit* mutant lacks the third class B region of the EGF1 domain, which includes six major Cys residues and five His residues, suggesting that BmVgR can bind with its ligands but cannot be dissociated under acidic conditions. These results for the functional domains of the *vit* mutational BmVgR could be consistent with earlier results.

The RT-PCR and *in situ* hybridization results showed that *BmVgR* was expressed only in oocytes, a finding consistent with results for other insects. For instance, VgR was expressed specifically in the insect ovary, including those of *Periplaneta americana* (9), *Leucophaea maderae* (11), *Blattella germanica* (10), *Drosophila melanogaster* (48), *Anopheles aegypti* (7), *Solenopsis invicta* (8), and *S. litura* (13). In addition, as *BmVg* starts to be expressed, the expression level of *BmVgR* is up-regulated, which is consistent with the timing of egg formation and maturation in the silkworm. The expression of *BmVg* is stimulated by ecdysone (49, 50), suggesting that the expression of *BmVgR* is also promoted by this hormone. Earlier studies implied that LDLRs can cross-react with other types of receptors in terms of their ligand recognition and immunoreactivity. For example, the chicken VgR has been shown to import very low-density lipoprotein and Vg into growing oocytes (21, 51). Tufail *et al.* (5) suggested that insect VgRs also recognize multiple ligands. Given that *vit* mutant eggs lack Vn as well as 30-kDa proteins, it might be that BmVgR can transport BmVg and 30-kDa proteins and even other lipid proteins that supply nutrition for ovarian development. Multiple forms of VgR were reported in tilapia (*Oreochromis aureus*) and white perch (*Morone americana*) (52, 53). Reading *et al.* (53) reported binding of multiple types of Vtg to the multiple forms of VtgR in vertebrates. BmVgR is expressed throughout the lifespan of the silkworm, which might have multiple types to bind other ligand proteins in addition to BmVg and 30-kDa proteins. These issues need confirmation by further experiments.

Although the *BmVgR* gene in the *vit* mutant has a 228-bp genomic deletion, it was transcribed in *vit* individuals, and its expression level was not reduced significantly when compared with that of the wild type. Therefore, the phenotype of the *vit* mutant is likely caused by BmVgR protein dysfunction. Linkage analysis also confirmed that the abnormality of *BmVgR* is responsible for the *vit* mutant phenotype. We further confirmed that the mutated BmVgR with a deletion of the third class B region in the EGF1 domain could bind ligands, for example, BmVg and 30-kDa proteins (data not shown), but that ligands cannot be dissociated under acidic conditions by co-IP. This suggests that BmVg, 30-kDa proteins, and even other ligands of the *vit* mutant can be transported into the ovary by mutational BmVgR-mediated endocytosis but cannot be dissociated under acidic conditions, which were found in the silkworm egg (23, 44, 47). The results showed that the defective BmVgR could lead to production of the *vit* mutant phenotype.

After RNAi silencing of *BmVgR* in the silkworm, the *BmVgR* signal is markedly reduced. In addition, the signal in moths that did not produce eggs (dsBmVgR-2) was lower than that of moths that produced small eggs (dsBmVgR-1). Therefore, silencing of the *BmVgR* gene was more efficient in moths that did not produce eggs (dsBmVgR-2). BmVn was also deficient in small dsBmVgR-treated eggs. Thus, nutritional shortages, such as BmVn and 30-kDa proteins, could lead to the formation of white, smaller eggs or none. This is similar to the phenotype recorded after RNAi in *B. germanica* (10), *Hemaphysalis longicornis* (12), *S. invicta* (54), *S. litura* (13), and similar to the phenotype of the *vit* mutant eggs that are white and smaller compared with WT eggs and homozygous lethal. These data further indicate that deficiency of BmVgR also results in the phenotype of the *vit* mutant. Similarly, the chicken *VgR* mutation leads to non-egg-laying sterility of *restricted ovulator* (R/O) (55). Deficient VgR is responsible for the female *D. melanogaster* sterility mutant *yolkless* (*yl*) (6). The requirement of BmVgR for egg formation suggests that ligands such as BmVg and 30-kDa proteins are also essential for egg formation and embryonic development in the silkworm. A defective or deficiency of BmVgR and the deficiency of yolk proteins, such as BmVg or 30-kDa proteins, could both lead to an insufficient nutrition supply for ovary development. Yamashita and Irie (27) reported that development of ovaries into mature eggs with chorion in the male silkworm and development of these eggs into larvae by artificial parthenogenesis could be explained by the fact that the male silkworm can synthesize BmVg and 30-kDa proteins in the fat body. Then, these proteins are secreted in the hemolymph and transported into the transplanted ovary by BmVgR-mediated endocytosis (data not shown).

Therefore, BmVgR has an important role in transporting yolk proteins for egg formation and embryonic development of the silkworm. The silkworm, *B. mori*, is a model member of the Lepidoptera, a taxon that includes many kinds of agricultural pests. Thus, BmVgR is a potential gene or protein target for pest control.

## Footnotes

4 The abbreviations used are:

Vn: vitellin
Vg: vitellogenin
VgR: vitellogenin receptor
LDLR: LDL receptor
LBD: ligand-binding domain
Bm: *Bombyx mori*
YPR: YP receptor
Ab: antibody
co-IP: coimmunoprecipitation
DIG: digoxigenin
qRT-PCR: quantitative RT-PCR.
